# Long-Term Antibody Response against SARS-CoV-2 in Health Care Workers: Effectiveness of Homologous and Heterologous Regimens and Their Relation to Systemic Vaccine-Associated Symptoms

**DOI:** 10.3390/vaccines10101599

**Published:** 2022-09-23

**Authors:** Helene Kierkegaard, Birgit Thorup Røge, Amanda Nissen, Jonna Skov Madsen

**Affiliations:** 1 Department of Biochemistry and Immunology, Lillebaelt Hospital, University Hospital of Southern Denmark, 6000 Kolding, Denmark; 2 Department of Internal Medicine, Lillebaelt Hospital, University Hospital of Southern Denmark, 6000 Kolding, Denmark; 3 Department of Regional Health Research, Faculty of Health Sciences, University of Southern Denmark, 5230 Odense, Denmark

**Keywords:** COVID-19 vaccines, vaccine effectiveness, BNT162b2 vaccine, mRNA-1273 vaccine, ChAdOx1 vaccine, 19 Elecsys Anti-SARS-CoV-2 S assay, reactogenicity, vaccine-associated symptoms

## Abstract

This prospective study provides data on the long-term humoral immunogenicity of a heterologous off-label vaccine regimen combining the adenoviral-vectored ChAdOx1 nCoV-19 from Astra-Zeneca (ChAd) with the mRNA-1273 vaccine from Moderna (m1273) in comparison with two different homologous mRNA vaccine schedules. Of the 316 COVID-19 naïve adult health care workers (HCW) included to complete a survey on vaccine-associated symptoms (VAS), 197 had received the homologous BNT162b2 mRNA vaccine from Pfizer/BioNTech (BNT/BNT), 76 the homologous m1273/m1273, and 43 the heterologous ChAd/m1273 vaccine regimen. The concentration of antibodies against SARS-CoV-2 spike protein in plasma 5–7 months after the second vaccine dose was higher in the m1273/m1273 and ChAd/m1273 than the BNT/BNT vaccine group. The frequency of systemic VAS after the first vaccine dose was 86% after ChAd compared with 35% and 39% after BNT and m1273, respectively (*p* < 0.0001), and after the second vaccine dose, the highest (89%) in the m1273/m1273 group (*p* < 0.001). Individuals with systemic VAS achieved higher levels of antibodies irrespective of vaccine regimen. In conclusion, VAS serve as a strong predictor of long-term humoral immune response, and the heterologous ChAd/m1273 vaccine regimen provides an at least equal long-term humoral immune response compared with the standard vaccine regimens used in Denmark.

## 1. Introduction

Vaccination against SARS-CoV-2 plays a major role in controlling the SARS-CoV-2 pandemic. Front-line health care workers (HCW) were prioritized to be among the first to receive the primary vaccines in Denmark, in addition to those at high risk of severe disease and death due to COVID-19. At the turn of the year 2020, the BNT162b2 mRNA vaccine from Pfizer/BioNTech (Pfizer or BNT) was put into use, followed by the introduction of the mRNA-1273 vaccine from Moderna (Moderna or m1273) on 14 January 2021. The viral vector ChAdOx1 vaccine from AstraZeneca (AstraZeneca or ChAd) was then introduced 25 days later on 9 February and used until 11 March 2021, at which time this vaccine was excluded from the Danish vaccination program due to rising concerns of Vaccine-Induced Immune Thrombosis and Thrombocytopenia (VITT) [1]. Thus, no one received a second dose of the ChAd vaccine at our hospital. According to recommendations from the Danish Health Authority in April 2021, individuals who had received a single ChAd vaccine dose were to be offered a second vaccine dose with one of the two used mRNA vaccines. For this purpose, our hospital used the m1273 vaccine, leaving a proportion of HCWs to receive the heterologous vaccine schedule ChAd/m1273.

Concerns were soon raised about waning vaccine immunity and the safety of heterologous schedules. A large Danish population-based study found a reduced risk of SARS-CoV-2 infection in individuals who had received the heterologous ChAd/m1273 vaccine schedule compared with unvaccinated individuals, which provided evidence for its clinical effectiveness [2]. However, the study also stated the need for a longer follow-up time as well as longitudinal data on the duration of vaccine-induced antibody levels obtained by this heterologous and other vaccine regimens [2]. A randomized study investigating the safety and immunogenicity of heterologous schedules with the ChAd and BNT vaccines found that the SARS-CoV-2 anti-spike IgG concentrations measured 28 days after the second dose were higher than what was obtained with the homologous ChAd/ChAd schedule. The heterologous ChAd/BNT showed a lower SARS-CoV-2 anti-spike IgG concentration compared with the homologous BNT/BNT regimen [3], whereas Schmidt et al. found that the same antibody level was obtained when comparing the BNT/BNT and the ChAd/BNT schedules [4].

A recent comprehensive review provides data on vaccine effectiveness for five homogeneous and four heterologous COVID-19 vaccine regimens and offers solid evidence for the clinical effectiveness of the homologous vaccine regimens, especially the mRNA vaccines [5]. However, data on most of the heterologous regimens are sparse, and the review does not include information on the ChAd/m1273 regimen used at our hospital [5]. This calls for more data on the safety and effectiveness of these heterologous vaccine regimens, as well as the fact they have been applied by several countries in response to changing recommendations and vaccine shortages. In addition to the call for further studies on other, new or updated vaccine regimens, there is also a need to evaluate waning immunity in different vaccine regimens and to determine which factors might predict vaccine effectiveness. Consequently, a number of studies have proposed and addressed an association between vaccine-associated symptoms and vaccine effectiveness. Here, adverse reactions after the homologous mRNA vaccines BNT/BNT and m1273/m1273 were associated with higher antibody levels in some [6,7,8,9] but not all studies [10]. No such association was found for the homologous ChAd/ChAd vaccine in a prospective observational study [11].

The primary aim of this prospective study was to determine and compare antibody levels obtained by three different vaccine regimens used in HCWs in a Danish medical center; the two homologous mRNA vaccines from Pfizer (BNT/BNT) or Moderna (m1273/m1273) and the heterologous AstraZeneca/Moderna (ChAd/m1273) vaccine regimen. The secondary aim was to investigate the association between systemic vaccine-associated symptoms and long-term antibody response.

## 2. Materials and Methods

### 2.1. Study Population and Design

In this prospective study, we recruited HCWs employed at Lillebaelt Hospital, University Hospital of Southern Denmark to participate in a survey on vaccine-associated symptoms (VAS) via the hospital’s internal website. Online recruitment was initiated on 25 February 2021. In addition, we consecutively invited the first 115 volunteers with no history of PCR-verified COVID-19 to participate in an antibody sub-study to determine long-term antibody response.

**Figure 1:**
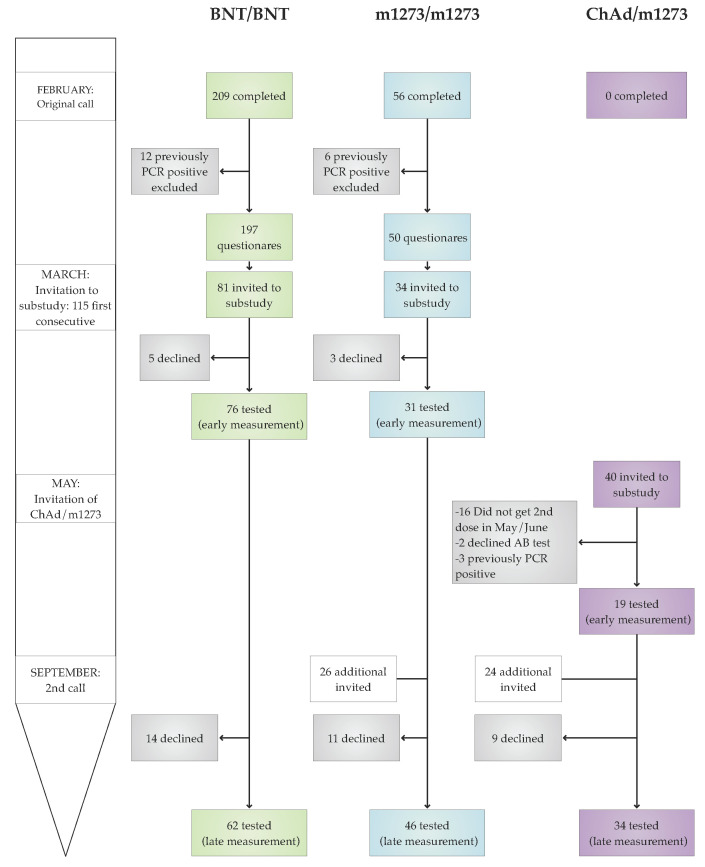
Outline of the study flow illustrating the timeline for recruiting participants according to vaccine regimens. Green: BNT/BNT, the BNT162b2 mRNA vaccine from Pfizer/BioNTech. Blue: m1273/m1273, the mRNA-1273 vaccine from Moderna. Purple: ChAd/m1273, the heterologous vaccine regimen combining ChAdOx1 vaccine from AstraZeneca with m1273 from Moderna. Early and late measurement refers to antibody level on average one and six months after completion of a vaccine regimen with a second vaccine dose, respectively.

The heterologous AstraZeneca/Moderna (ChAd/m1273) vaccine regimen was introduced in April 2021 for those HCWs who had received their first vaccine dose with the ChAd vaccine. Thus, in May 2021, there was an additional call to recruit individuals from this heterologous vaccine group. Finally, as the homologous Pfizer (BNT/BNT) was by far the most prominent vaccine regimen used, a last call was announced in September 2021 to recruit more individuals in the heterologous AstraZeneca/Moderna (ChAd/m1273) and the homologous Moderna (m1273/m1273) vaccine regimen groups. The last blood sample was collected on 2 December 2021. The study outline and flow are presented in Figure 1.

Participants completed a structured questionnaire on systemic and local VAS, vaccine regimen, and known pre-existing co-morbidities in addition to information on any positive PCR-test for SARS-CoV-2, both before and after the first vaccine dose and during the entire follow-up period until the late antibody measurements. The systemic VAS were defined as chills, fever, fatigue, muscle pain, headache, or nausea. The full-length questionnaire is provided in Supplementary Table S1.

Blood samples for early and late antibody response were drawn 1 and 6 (+/−1) months after the second vaccine dose, centrifuged at 2000× *g* for 10 min. Plasma was transferred to cryotubes and stored at −80 °C until analysis.

### 2.2. SARS-CoV-2 Antibody Measurement

The quantitative measurements of antibodies against SARS-CoV-2 spike (S) protein in all plasma samples were analyzed blinded to clinical data using Elecsys® Anti-SARS-CoV-2 S immunoassay on the Cobas e 801 platform (Roche Diagnostics GmbH, Germany). The immunoassay uses a recombinant protein representing the receptor-binding domain (RBD) of the S antigen. According to the manufacturer, the unit U/mL for the Elecsys Anti-SARS-CoV-2 S assay is equivalent to the Binding Antibody Unit (BAU/mL) for the First WHO International Standard for anti-SARS-CoV-2 immunoglobulin [12]. The measuring range for the assay is 0.4 U/mL to 2500 U/mL, and values above 0.8 U/mL are interpreted as positive. All analyses were carried out at the Department of Biochemistry and Immunology, Lillebaelt Hospital, which is accredited by the Danish Accreditation Fund (DANAK) according to the ISO 15189:2012 standard that specifies requirements for quality and competence in medical laboratories.

### 2.3. Statistical Analysis

Originally, this study was powered to detect differences in antibody level between the three homologous vaccine regimens; BNT/BNT, m1273/m1273, and ChAd/ChAd. Calculations were based on assumptions, as no literature on the subject had been published at the initiation of the study. Calculations of sample size based on one-way Anova with a large effect size (0.4), a significance level of 0.05, and a power of 0.90 resulted in 28 persons per group. We therefore invited HCWs consecutively until at least 30 consented in each group. As illustrated in Figure 1, due to the discontinuation of the ChAd/ChAd regimen, 40 HCWs were invited in May in the ChAd/m1273 group but only 19 accepted antibody measurements. Post hoc power analysis with only 19 in each group revealed a power of 0.75. We chose to invite additional participants in both the m1273/m1273 and ChAd/m1273 groups to increase power at the late measurement.

Values are reported as medians with ranges or interquartile ranges (IQR) and n (%). Differences between groups were tested with the Kruskal–Wallis non-parametric test or the Chi-Square test. The odds ratio was adjusted for age by logistic regression, and the presence of systemic VAS was correlated with the level of antibodies at the late measurement 6 months after completion of the vaccine regimen with a second vaccine dose. The graphical presentations and data analysis were performed using SAS® Enterprise Guide, Version 7.15, SAS Institute Inc. Cary, NC, USA and R Core Team (2021). R: A language and environment for statistical computing. R Foundation for Statistical Computing, Vienna, Austria. URL https://www.R-project.org/ accessed on 1 February 2022. A *p* value < 0.05 was considered statistically significant.

## 3. Results

### 3.1. Study Participants and Vaccine Regiments

The online questionnaire was answered by 337 health care workers, of which 209 received the Pfizer (BNT/BNT), 82 the Moderna (m1273/m1273), and 46 the AstraZeneca/ Moderna (ChAd/m1273) vaccine regimens. Of these, 12 individuals in the BNT/BNT, 6 in the m1273/m1273, and 3 the ChAd/m1273 group reported PCR-confirmed SARS-CoV-2 infection prior to the first vaccine dose. No one reported PCR-confirmed SARS-CoV-2 infection between the first and second vaccine doses, leaving 197, 76, and 43 for the data analyses of VAS, respectively.

The timespan between the first and second vaccine doses for the homologous vaccine regimens followed the manufacturers’ recommendations. No such recommendation exists for the heterologous vaccine regimen, which was awaiting government approval to be introduced. Thus, the timespan between the first and second vaccine doses was longer in the heterologous ChAd/m1273 vaccine group than in the two homologous vaccine groups. The median timespan between the first and second vaccine doses is 85 days (range, 40–85) in the ChAd/m1273 vaccine group compared with 28 (20–60) and 36 (29–43) days in the BNT/BNT and m1273/m1273 vaccine groups, respectively. Therefore, individuals in the heterologous ChAd/m1273 vaccine group completed the vaccine program later, and the average time between early and late antibody measurements was shorter than it was for individuals receiving one of the two homologous vaccine regimens. The diagram for the study flow is presented in Figure 1, and Table 1 provides the characteristics of study population according to vaccine regimen together with vaccine-associated symptoms.

### 3.2. Vaccine-Associated Symptoms

The frequency of reported systemic symptoms after the first vaccine dose was 86% in individuals receiving the AstraZeneca (ChAd) vaccine compared with 35% and 39% in those receiving the Pfizer(BNT) and Moderna(m1273) vaccine, respectively (*p* < 0.0001). In pairwise comparison, no difference in VAS between Pfizer and Moderna was found (*p* = 0.49), whereas the difference in VAS between both the Pfizer and AstraZeneca vaccine and the Moderna and AstraZeneca vaccine were highly significant (for both pairwise comparisons *p* < 0.000001). However, the frequency of systemic VAS after the second vaccine dose was the highest in those receiving the homologous Moderna (m1273/m1273) vaccine regimen compared with those receiving either the homologous Pfizer (BNT/BNT) or the heterologous AstraZeneca/Moderna (ChAd/m1273) vaccine regimen, overall *p*-value < 0.001. Data on the frequency of vaccine-associated symptoms are presented in Table 1. Preexisting co-morbidity showed no association with systemic VAS (Chi-Square test *p* = 0.58, data not shown).

### 3.3. Early and Late Antibody Level According to Vaccine Regimen

**Figure 2:**
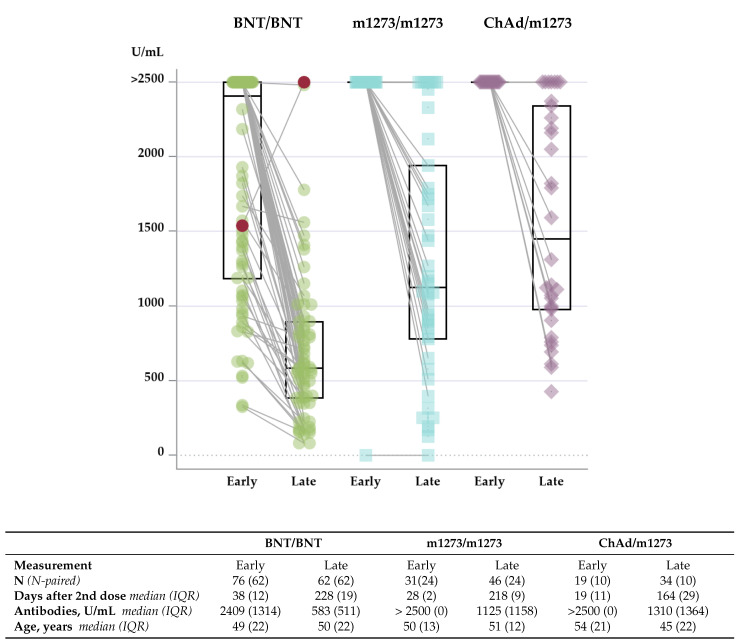
Concentration of antibodies against SARS-CoV-2 spike (S) protein in plasma at late and early measurements according to vaccine regimen. Results are presented as box plots, where the ends of the boxes define the 25th and 75th percentiles and with a line at the median. Individual values for each vaccine group appear as green circles (BNT/BNT, the BNT162b2 mRNA vaccine from Pfizer/BioNTech), blue squares (m1273/m1273, the mRNA-1273 vaccine from Moderna), and purple diamonds (ChAd/m1273, the heterologous vaccine regimen combining ChAdOx1 vaccine from AstraZeneca with m1273 from Moderna). Individuals with paired measurements (both early and late antibody measurements in the same person) are marked with a line combining the two measurements. N is the number of individuals in each vaccine group and N-paired is the number of individuals with paired measurements. The red circle indicates one participant with a PCR-confirmed infection with SARS-CoV-2 between early and late tests. Interquartile range (IQR) indicates the difference between the 75th and 25th percentile.

After we excluded individuals who reported having a positive PCR test for SARS-CoV-2 prior to the first vaccine dose, blood samples for antibody measurement were available from a total of 172 individuals (160 females) within the age range of 24–67 years. Data on early antibody levels measured median 31 (IQR 12) days after the second vaccine dose were available from 126 individuals, whereas data on late antibody levels measured median 218 (IQR 21) days after the second vaccine dose were available from 142 individuals and combined early and late antibody levels from 96 individuals. Early and late antibody levels according to vaccine regimen are presented in Figure 2.

The early level of antibody obtained was higher in individuals receiving the homologous Moderna(m1273/m1273) or the heterologous AstraZeneca/Moderna(ChAd/m1273) vaccine regimen than in those who received the Pfizer(BNT/BNT) vaccine regimen. Thus, at the early measurement, all individuals except one in both the m1273/m1273 and the ChAd/m1273 vaccine group obtained levels of antibodies above 2500 U/mL, which is the upper measuring range for the assay. In the BNT/BNT vaccine group, the median (IQR) antibody concentration was 2409 (1314) U/mL. The Kruskal–Wallis non-parametric test of the difference between the three groups was highly significant, with an overall *p*-value < 0.0001, BNT/BNT vs m1273/m1273, *p* < 0.0001, and BNT/BNT vs ChAd/m1273, *p* = 0.0004. This difference in antibody level between the m1273/m1273 or ChAd/m1273 groups and the BNT/BNT vaccine group remained at the late measurement 5–7 months after completion of the vaccine regimen with the second dose. No significant difference in antibody levels between the m1273/m1273 and the ChAd/m1273 vaccine group could be demonstrated at either early or late measurements.

**Figure 3:**
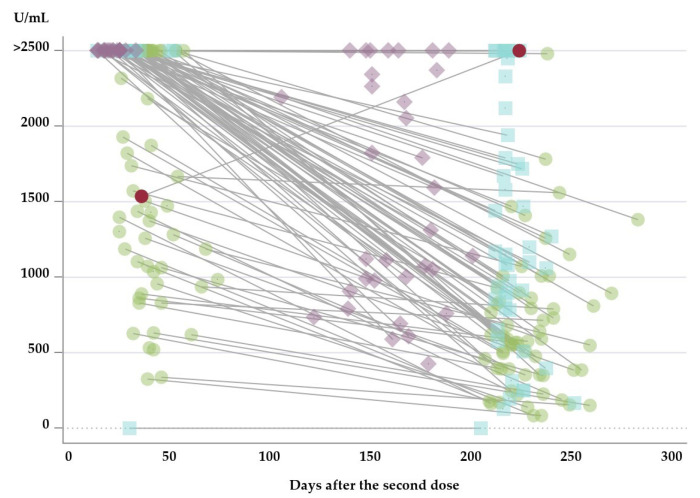
Concentration of antibodies against SARS-CoV-2 spike (S) protein in plasma after completion of the vaccine regimen with the second dose of vaccine. Green circles: BNT/BNT, two doses BNT162b2 mRNA vaccine from Pfizer/BioNTech). Blue squares: m1273/m1273, two doses mRNA-1273 vaccine from Moderna. Purple diamonds: ChAd/m1273, the heterologous vaccine regimen combining ChAdOx1 vaccine from AstraZeneca with m1273 from Moderna. Paired measurements are marked with lines. The individual marked with red had a PCR-confirmed infection with SARS-CoV-2 between the early and late antibody measurements.

Regardless of the vaccine regimen received, the 96 paired dataset demonstrated lower antibody levels at late compared with early measurements in all groups (*p* < 0.001). No difference in the waning of antibodies could be demonstrated between groups (*p* = 0.44, data not shown). Figure 3 outlines the dynamic of antibody level over time after completion of the vaccine regimen. One participant in the BNT/BNT vaccine group who had a PCR-confirmed break-through infection 5 months after the second dose of BNT vaccine achieved an antibody concentration above the upper measuring limit at the late measurement. In addition, in the m1273/m1273 vaccine group, one participant with known medical-treated autoimmune disease did not achieve a measurable humoral immune response. This is illustrated in Figure 2 and Figure 3.

### 3.4. Vaccine-Associated Symptoms Predict Late Antibody Level

**Figure 4:**
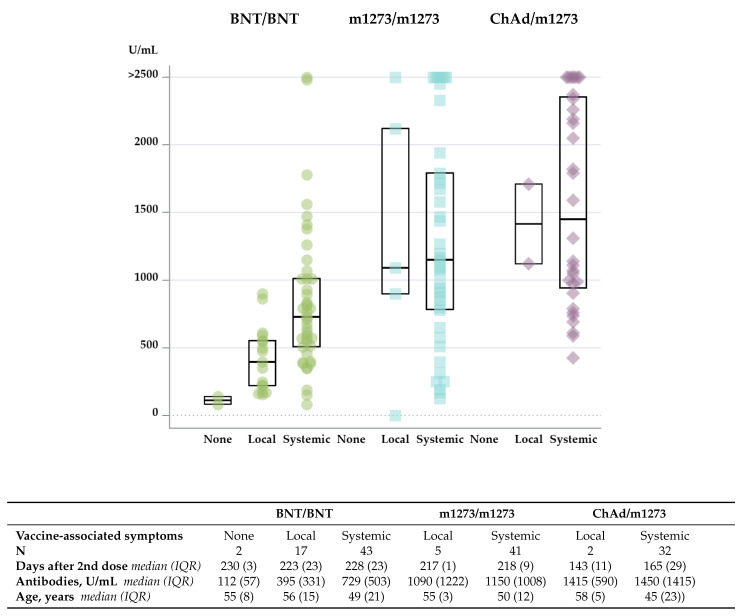
Concentration of antibodies against SARS-CoV-2 spike (S) protein in plasma 5 to 7 months after completion of vaccine regimen with the second dose of vaccine according to vaccine regimen and vaccine-associated symptoms (VAS). Results are presented as both individual values and as box plots, where the ends of the boxes define the 25th and 75th percentiles, and with a line at the median. Green circles: BNT/BNT, the BNT162b2 mRNA vaccine from Pfizer/BioNTech. Blue squares: m1273/m1273, the mRNA-1273 vaccine from Moderna. Purple diamonds: ChAd/m1273, the heterologous vaccine regimen combining ChAdOx1 vaccine from AstraZeneca with m1273 from Moderna. None refers to no VAS; Local refers to local VAS, and Systemic to systemic VAS. Interquartile range (IQR) indicates the difference between the 75th and 25th percentile.

HCWs with systemic VAS after any dose were younger than HCWs without systemic VAS (Kruskal–Wallis non-parametric test *p* = 0.011). Figure 4 illustrates that individuals with systemic VAS after any dose achieved a higher level of antibodies compared with those with only local or no VAS (*p* < 0.0001) and median ages for each group. The likelihood of having an antibody level above 700 U/mL measured at long-term follow up was associated with systemic VAS with an odds ratio (OR) of 5.42 (95% CI 2.15–13.67, *p* = 0.0002) and remained significant after adjusting for age. Thus, the age-adjusted OR was 5.15 (95% CI 2.00–13.25, *p* = 0.0008).

## 4. Discussion

In the current longitudinal study, we compare the long-term humoral response to three different vaccines against SARS-CoV-2 used in a cohort of SARS-CoV-2 naïve Danish HCWs. We provide evidence that both the effectiveness and durability of the humoral immune responses induced by the heterologous off-label AstraZeneca/Moderna (ChAd/m1273) vaccine regimen is at least equivalent to the homologous standard vaccine regimens with either Pfizer (BNT/BNT) or Moderna (m1273/m1273) used in Denmark. In addition, the antibody level 5 to 7 months after the second vaccine dose is higher in individuals with systemic VAS than in individuals with no or only local reactions/symptoms after vaccination. VAS as a predictor for vaccine effectiveness adds to the findings of other studies [6,7,8,9] and serves as reassuring knowledge when caring for patients experiencing VAS. Thus, a study in patients undergoing hemodialysis found that individuals with VAS obtained a stronger humoral immune response [13]. However, it was also found that 33% of the patients did not develop a substantial antibody response [13]. This highlights the clinical challenge that pre-existing comorbidity may diminish the likelihood of seropositivity after vaccination, recently addressed and demonstrated in several studies [14,15,16].

A comprehensive review on vaccine effectiveness according to vaccine regimen provides solid evidence for the clinical effectiveness of the homologous vaccine regimens, particularly the mRNA vaccines [5]. However, data for most of the heterologous regimens included in this paper were limited, with no data for the heterologous ChAd/m1273 vaccine regimen [5]. This calls for additional data on the clinical effectiveness and safety of heterologous vaccine regimens, especially since several countries have applied heterologous vaccine regimens in response to changing recommendations and vaccine shortages. There is also a need for longitudinal data on the duration of vaccine-induced antibody levels to provide information on both the level and duration of protecting antibodies obtained by different vaccine schedules.

In our study, the early and late measurement of antibodies against SARS-CoV-2 spike (S) protein in plasma took place 1 and 5–7 months after the second vaccine dose completing the primary vaccine regimen. We found the early antibody concentration to be significantly higher in individuals receiving the homologous Moderna (m1273/m1273) or the heterologous AstraZeneca/Moderna (ChAd/m1273) vaccine regimen than in those receiving the homologous Pfizer (BNT/BNT) vaccine. In comparison, a randomized study investigating the safety and immunogenicity of heterologous schedules with the ChAd and BNT vaccines found that the obtained antibody concentrations of both heterologous schedules measured 28 days after the second dose were higher than with the homologous ChAd/ChAd schedule. However, they found that antibody concentration was lower in the heterologous ChAd/BNT than in the homologous BNT/BNT regimen [3], which contradicts what we found in our heterologous ChAd/m1273 vaccine regimen compared with the homologous BNT/BNT regimen. A study by Schmidt et al. found similar antibody levels 2 weeks after the second vaccine dose when comparing homologous mRNA vaccine regimens (BNT162b2 or mRNA-1273) and heterologous ChAd/mRNA vaccine schedules. This study, however, does not distinguish between the two mRNA vaccines (BNT162b2 or mRNA-1273) in the data analysis [4].

The long-term humoral response in our study was assessed by the concentration of antibodies against SARS-CoV-2 spike (S) protein 5–7 months after the completion of the vaccine regimen with the second vaccine dose. With this long-term follow up, we found that the antibody concentration in individuals receiving the homologous Moderna (m1273/m1273) or the heterologous AstraZeneca/Moderna (ChAd/m1273) vaccine regimen was higher than that of those receiving the homologous vaccine regimens with Pfizer (BNT/BNT). Furthermore, by mutual comparison, the concentration of antibodies was higher in individuals receiving the heterologous ChAd/m1273 compared with the homologous m1273/m1273 vaccine regimen. This, however, we attribute to be a consequence of the waning of antibodies over time, where, in individuals receiving the heterologous ChAd/BNT vaccine regimen, the antibody level was measured median 164 days after the second vaccine dose compared with 218 and 228 days in those receiving the homologous m1273/m1273 or BNT/BNT vaccine regimen, respectively. Therefore, we do not consider that our results allow concluding a superiority in long-term humoral immune response for the heterologous ChAd/m1273 vaccine regimen. However, it provides an at least equal long-term humoral immune response compared with the two homologous standard vaccination regimens, BNT/BNT and m1273/m1273, used in Denmark.

A recent study from Greece provide data on a homologous ChAd/ChAd vaccine regimen, comparing the long-term antibody response of this regimen with the homologous BNT/BNT vaccine regimen, and found the BNT/BNT vaccine to be superior with higher levels of antibodies at both 3 and 6 months post-vaccination [17]. Similarly, a short-term study comparing these two vaccine regimens (BNT/BNT and ChAd/ChAd) found higher antibody concentrations 2 weeks after the second vaccine dose in the BNT/BNT comparedwith the ChAd/ChAd vaccine group [18]. Thus, in these two studies, the homologous BNT/BNT vaccine regimen obtained a higher long-term antibody level than the homologous ChAd/ChAd vaccine regimen [17,18], whereas in our study, the ChAd vaccine, when part of the heterologous ChAd/m1273 vaccine regimen, performed better than the homologous BNT/BNT vaccine regimen. Other recent studies also suggest that the heterologous vaccine regimens have the advantage of achieving a protective level of antibodies. Thus, a study with 84 days of follow-up shows superior antibody immunity induced by the heterologous ChAd/m1273 than the homologous ChAd/ChAd vaccine regimen [19]. In addition, a recent cross-sectional and uncontrolled study provide further evidence to support a long-term antibody response obtained by the heterologous AstraZeneca/Moderna vaccine regimen by reporting data on antibody levels 6 month after completing the vaccine regimen [20]. Furthermore, a study that measured both level of antibodies and memory B cell responses on the day of vaccine regimen completion with the second vaccine dose, and again after 7 to 9 days, found that individuals who received the heterologous ChAd/m1273 vaccine obtained a higher level of antibodies than those who received the homologous ChAd/ChAd vaccine regimen [21].

Several heterologous vaccine regimens are being applied in multiple countries in response to changing recommendations and vaccine shortages. This highlights the need for data on the effectiveness and safety of these regimens. In addition, there is a need for further studies to evaluate waning immunity in other, new or updated vaccine regimens and to determine the factors that might predict this. With our study, we contribute to knowledge in this area with data on long-term humoral immune response following two homologous and one heterologous vaccine regimens. Still, data on heterologous vaccine regimens are especially sparse and warranted. In addition, we provide detailed data on the relation between VAS and levels of antibodies. A strength of our study is the prospective longitudinal design, ensuring good quality of the data. A clear limitation, however, is the relatively small number of individuals included in our heterologous ChAd/m1273 vaccine group. In addition, our study does not investigate the cellular immune response to vaccination but only the humoral counterpart. Therefore, the vaccine effectiveness in our study is based only on the long-term humoral response.

## 5. Conclusions

Vaccine-associated symptoms are a strong predictor of vaccine effectiveness based on long-term humoral response. The heterologous AstraZeneca/Moderna (ChAd/m1273) vaccine regimen provides an at least equal long-term humoral immune response compared with the two homologous standard vaccine regimens, Pfizer (BNT/BNT) or Moderna (m1273/m1273), used in Denmark. Future studies are needed to provide data on the effectiveness and safety of each vaccine regimen. In this context, it seems especially important to investigate vaccine effectiveness in those who may need it the most. In addition, there is a need to investigate what may predict vaccine effectiveness to define who will need a booster vaccine does and when it is necessary. Results from such studies will help develop appropriate guidelines for booster doses and targeted vaccination programs.

## Figures and Tables

**Table 1:** Characteristics of the cohorts according to vaccine regimen.

	BNT/BNT	m1273/m1273	ChAd/m1273	*p*-Value ^1^
Answers on questionnaire, n	209	82	46	
PCR-confirmed SARS-CoV-2 infection prior to first vaccine dose, n (%) (PP)	12 (6%)	6 (7%)	3 (7%)	0.88
PCR-confirmed SARS-CoV-2 during follow up, n	1	0	0	
**Study cohort excluding individuals with PCR-confirmed SARS-CoV-2 infection prior to first vaccine dose**				
**Demographics**				
HWCs (N)	197	76	43	
Sex: female (%)	185 (94%)	70 (92%)	39 (90%)	0.70
Age: median (range), years	48 (19–67)	50 (21–67)	45 (24–64)	0.56 *
Preexisting co-morbidity	48 (24%)	23 (30%)	9 (21%)	0.47
**Vaccine-associated symptoms**				
*Systemic symptoms*				
After the first dose	69 (35%)	30 (39%)	37 (86%)	<0.0001
After the second dose	127 (64%)	68 (89%)	33 (77%)	<0.001
After any dose	140 (71%)	69 (91%)	42 (98%)	<0.0000001
*Local symptoms*				
After the first dose	162 (82%)	71 (93%)	36 (84%)	0.06
After the second dose	137 (70%)	71 (93%)	33 (80%)	<0.001
After any dose	174(88%)	75(99%)	38(93%)	0.02
Any VAS after any dose	191 (97%)	76 (100%)	43 (100%)	0.16

^1^ All *p*-values are calculated with the Chi-Square test, except for the number marked with ^*^ where the nonparametric Kruskal–Wallis test is used. BNT/BNT: the BNT162b2 mRNA vaccine from Pfizer/BioNTech. m1273/m1273: the mRNA-1273 vaccine from Moderna. ChAd/m1273: the heterologous vaccine regimen combining the ChAdOx1 vaccine from AstraZeneca with m1273 from Moderna. VAS: vaccine-associated symptoms. HWCs: health care workers. Sex: biological sex.

## Data Availability

Not applicable.

## Supplementary Materials

The following supporting information can be downloaded at: https://www.mdpi.com/article/10.3390/vaccines10101599/s1, Table S1: Online Questionnaire.

## Abbreviations

The following abbreviations are used in this manuscript:

VAS	Vaccine-associated symptoms
BNT	BNT162b2 mRNA vaccine from Pfizer/BioNTech
ChAd	ChAdOx1 nCoV-19 from AstraZeneca
m1273	mRNA-1273 vaccine from Moderna
SARS-CoV-2	Severe acute respiratory syndrome coronavirus 2
HCW	Health care workers
VITT	Vaccine-Induced Immune Thrombosis and Thrombocytopenia
COVID-19	Coronavirus disease 2019
