# Supplementary material for: Long-Term Antibody Response against SARS-CoV-2 in Health Care Workers: Effectiveness of Homologous and Heterologous Regimens and Their Relation to Systemic Vaccine-Associated Symptoms
Source: Vaccines (Basel). 2022 Sep 23;10(10):1599. doi: 10.3390/vaccines10101599 (PMC9611514; doi:10.3390/vaccines10101599)
Supplement: Supplementary file 1 [file vaccines-10-01599-s001.zip › vaccines-1908308-supplementary.pdf]

Table S1. Online Questionnaire

No.	Question	Answer	Sub-answer
1a	Have you previously tested positive for SARS-CoV-2?	Yes/no	If yes, date (yyyy-mm-dd)
2a	What vaccine did you get?	Comirnaty® (Pfizer)<br>COVID-19 vaccine Moderna®<br>COVID-19 vaccine AstraZeneca®<br>Do not know<br>Do not wish to tell	
2b	Date of 1st vaccine dose	yyyy-mm-dd	
2c	Date of 2nd vaccine dose	yyyy-mm-dd	
3	Vaccine-associated symptoms after 1st vaccine dose		
3a	Headache*	Yes/no	If yes, duration in days
3b	Muscle pain*	Yes/no	If yes, duration in days
3c	Feeling of fever / chills*	Yes/no	If yes, duration in days
3d	Nausea*	Yes/no	If yes, duration in days
3e	Fatigue*	Yes/no	If yes, duration in days
3f	Dizziness*	Yes/no	If yes, duration in days
3g	Sore arm	Yes/no	If yes, duration in days
3h	Other	Yes/no	If yes, describe
4	Vaccine-associated symptoms after 2nd vaccine dose		
4a	Headache*	Yes/no	If yes, duration in days
4b	Muscle pain*	Yes/no	If yes, duration in days
4c	Feeling of fever / chills*	Yes/no	If yes, duration in days
4d	Nausea*	Yes/no	If yes, duration in days
4e	Fatigue*	Yes/no	If yes, duration in days
4f	Dizziness*	Yes/no	If yes, duration in days
4g	Sore arm	Yes/no	If yes, duration in days
4h	Other	Yes/no	If yes, describe
5	Do you have a chronic illness?	Yes/no	If yes, describe
6	May we contact you for further research?	Yes/no	

* Vaccine-associated symptoms marked with a star were grouped as systemic. Sub-answers were only shown if the answer before was "yes".
